# Motion-based tissue *ex vivo* (MOTEX) assay to assess proton and X-ray irradiation responses in head and neck squamous cell carcinoma

**DOI:** 10.1016/j.ctro.2026.101124

**Published:** 2026-02-08

**Authors:** Katrin S. Pachler, Iris Lauwers, Nicole S. Verkaik, Marta Rovituso, Hetty Mast, Brend P. Jonker, Bernd Kremer, Sjors A. Koppes, Thierry P.P. van den Bosch, Gerda M. Verduijn, Steven Petit, Brita S. Sørensen, Dik C. van Gent, Marta E. Capala

**Affiliations:** a Department of Molecular Genetics, Erasmus MC, P.O. Box 2040, 3000 CA Rotterdam, Netherlands (the); b Department of Radiotherapy, Erasmus MC Cancer Institute, P.O. Box 2040, 3000 CA Rotterdam, Netherlands (the); c Holland Proton Therapy Centre (HPTC), Huismansingel 4, 2629 JH Delft, Netherlands (the); d Department of Oral and Maxillofacial Surgery, Erasmus MC Cancer Institute, P.O. Box 2040, 3000 CA Rotterdam, Netherlands (the); e Department of Otorhinolaryngology and Head and Neck Surgery, Erasmus MC Cancer Institute, P.O. Box 2040, 3000 CA Rotterdam, Netherlands (the); f Department of Pathology, Erasmus MC, P.O. Box 2040, 3000 CA Rotterdam, Netherlands (the); g Department of Experimental Clinical Oncology, Danish Centre for Particle Therapy, Aarhus University Hospital, Palle Juul-Jensens Boulevard 99, DK-8200 Aarhus N, Denmark; h Oncode Institute, Jaarbeursplein 6, 3521AL Utrecht, Netherlands (the)

**Keywords:** HNSCC, Response prediction, Radiotherapy, *Ex vivo* assay, Homologous recombination deficiency (HRD), Tumor infiltrating lymphocytes

## Abstract

**Background and purpose:**

Proton radiotherapy is applied for various tumor sites, offering better dose distribution resulting in decreased excess radiation of healthy tissue. Furthermore, the biological effects of proton irradiation may be different from X-ray irradiation, depending on tumor characteristics. This is particularly relevant for head and neck squamous cell carcinoma (HNSCC), which displays high biological heterogeneity. However, this heterogeneous response to various radiation modalities is currently not included in clinical decision making due to lack of response prediction models.

**Materials and methods:**

Nine oral cavity tumor specimens were obtained after surgical resection, cut into thin slices, irradiated with both X-ray and protons, and cultured *ex vivo* for up to five days. We subsequently analyzed proliferation, apoptosis, DNA repair and immune cell infiltration.

**Results:**

Most tumors (five out of nine) showed similar response to proton and X-ray irradiation. However, in three tumors a significantly larger decrease in viability was measured upon proton irradiation. One of those tumors was homologous recombination deficient (HRD). The other two proton-sensitive tumors showed low numbers of infiltrating immune cells, including tumor infiltrating lymphocytes, while the most X-ray sensitive tumor had a particularly high immune cell infiltration.

**Conclusion:**

The *ex vivo* assay revealed heterogeneous response of HNSCC tumors to proton and X-ray irradiation. The tumors with increased sensitivity to proton irradiation either showed HR deficiency or low immune cell infiltration. However, not all immune-deserted tumors showed this skewing towards X-ray resistance. These findings require validation in a larger cohort of patients.

## Highlights

• Proton-sensitive HNSCC tumors were identified with an *ex vivo* irradiation.
• One of the proton-sensitive tumors was homologous recombination (HR) deficient.
• Two X rays-resistant but proton-sensitive tumors had low immune cell infiltration.

## Graphical abstract

**Figure:**
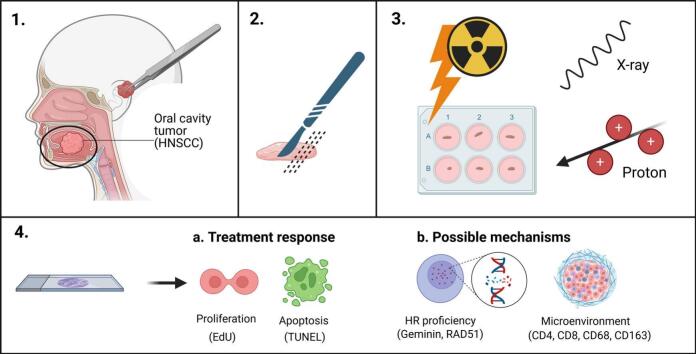

## Introduction

Surgical resection and (chemo-)radiotherapy are the most common treatment modalities for head and neck squamous cell carcinoma (HNSCC) [1]. The radiotherapy dose is limited for tumors in the pharynx and oral cavity area, due to surrounding vital structures, so-called organs-at-risks (OARs) [2]. Proton radiotherapy offers a favorable dose distribution and decreased excess radiation of healthy tissue in comparison to conventional X-ray radiotherapy [3], [4], [5], [6]. Treatment requires balancing sufficient radiation dose to the tumor against sparing of the OARs, which is hampered by the heterogeneity in tumor responses. As this high level of heterogeneity cannot be explained entirely by clinical factors [7], several biological properties of the tumor have been proposed to influence radiotherapy response [1], [2].

Repair of DNA double strand breaks (DSBs), the main cell-killing effect of ionizing radiation, has been investigated extensively. Mutations in DNA repair pathways rendered some patients more sensitive to irradiation [8]. The main DNA repair pathways that counteract radiation-induced DSBs are homologous recombination (HR) and non-homologous end joining (NHEJ). While NHEJ is important for repairing both X-ray and proton induced DNA damage, several studies reported that HR is relatively more important for the repair of DSBs caused by proton irradiation [8], [9]. Due to higher linear energy transfer (LET) in the Bragg peak, protons can cause more clustered and complex DNA damage than X-rays. HR may be required for repair of these complex DSBs [8], [9], suggesting that differences in sensitivity to various types of radiation might be explained by the relative efficiency of HR and NHEJ.

The heterogeneous response to radiotherapy in HNSCC patients could also be related to the immune system. In general, HNSCC tumors can be categorized as immune-infiltrated “hot” and immune-deserted or immune-excluded “cold” tumors, based on the abundance of immune cell invasion [3]. The “immune-cold” HNSCC tumors generally have a worse prognosis, highlighting the potential benefit of including HNSCC microenvironment characteristics in the treatment response prediction models [4], [5]. Furthermore, several studies suggest that the type of radiation influences HNSCC tumor microenvironment responses, which could also have an effect on the tumor control upon radiotherapy [6], [7].

Existing predictive models for patient-specific radiosensitivity all have important drawbacks. While mouse models and 3D tumor organoids lack the complex patient-specific immune system, sequencing studies often miss the functional validation [8], [10]. To overcome this problem, we have established a motion-based *ex vivo* tissue slice culture (MOTEX) model using precision cut tumor slices. This model sustains cancer cell and immune cell viability in culture over several days, enabling evaluation of functional treatment response fitting the clinical timeframe [9].

Here, we describe the analysis of HNSCC tissue slices treated with proton and X-ray irradiation to investigate the patient-specific sensitivity to both modalities and elucidate the underlying mechanisms.

## Materials and methods

### Collection of tumor tissue

Resection material was acquired from HNSCC patients, which were undergoing surgery at the Erasmus University Medical Center (Erasmus MC), The Netherlands, as described previously [9]. Samples were pseudonymized according to the code of proper secondary use of human tissue in the Netherlands established by the Dutch Federation of Medical Scientific Societies, and approved by the Erasmus MC Medical Ethical Committee (number MEC-2017-1049).

### Tissue slice culture and irradiation

Tumor tissue processing and culture have been performed as described previously [9], [11]. X-ray irradiation was performed at Erasmus MC in an X-Strahl RS320 X-ray cabinet with 0.5 mm Cu filter at 195 keV with a dose rate of 1.6 Gy/min. Proton irradiation was performed at Holland Proton Therapy Center (HPTC) in the R&D beam line. From a therapeutic pencil beam, a passive scattering system produced a field size of 8 × 8 cm^2^, with dose uniformity of 98% over the whole area, irradiating the whole sample uniformly with a dose rate of 9 Gy/min. To create a Spread-out-Bragg peak (SOBP), a 2D range energy modulator was used with an initial energy of 150 MeV, creating a SOBP of 25 mm with 98% ±1% uniformity [3]. A physical dose of 5 Gy was given (not RBE weighted). Irradiations were performed simultaneously at both locations. Fixation was undertaken at 2 h and 5 days with prior 2-hour EdU incubation (30 μmol/L) [11], [12].

### Immunohistochemistry (IHC) and Immunofluorescence (IF)

All IHC stainings were automated by using the Ventana Benchmark ULTRA (Ventana Medical Systems Inc.) as reported previously [11]. IF was done as described previously [9], [13]. In Situ Cell Death Detection Kit (Roche Life Sciences) was used for apoptosis staining (TUNEL) as described [13].

### Image acquisition

H&E and CD68 IHC staining (DAB) were visualized using an Olympus BX40 F4 System microscope. CD45 immunohistochemistry staining (DAB) was imaged with a Zeiss Axio Imager II with Axiocam 208 camera. TUNEL staining (at least three fields of view (FoV) per sample) and RAD51 staining (at least 30 Geminin-positive cells per sample) were imaged using a Leica DM4000B fluorescent microscope with a Leica DFC300 FX camera. A Leica Stellaris 5 LIA confocal microscope was used for EdU incorporation (at least three FoV per sample).

### Image analysis

To calculate apoptosis levels, nuclei were segmented on the DAPI images and on the TUNEL images using a previously described StartDist module in Python 3.8 [11]. To calculate the proliferation, segmentation of EdU-positive cells was done as described previously [11].

RAD51 foci were analyzed as described previously [9], [13].

For CD45 quantification, all images were analyzed in QuPath with positive cell detection and two separate thresholds (1st hematoxylin for nuclei, 2nd DAB for CD45).

### Multiplex immunofluorescence

Multiplex IF analysis was performed by automated IF using the Ventana Benchmark Discovery ULTRA (Ventana Medical Systems Inc.; Supplementary Material and Methods; Supplementary Table 1) [14]. Immune cell numbers were calculated as a percentage relative to the total cell number in the tumor area.

### Statistical analysis

Statistical analysis and graph generation were performed using GraphPad Prism 8.0. Statistical differences were tested with Mann Whitney U Test and Kruskal Wallis test with Dunn’s comparison (independent values) or Wilcoxon Test and Friedman Test (dependent values) and p-values < 0.05 were considered significant.

## Results

### Simultaneous X-ray and proton irradiation in MOTEX culture

**Fig. 1:**
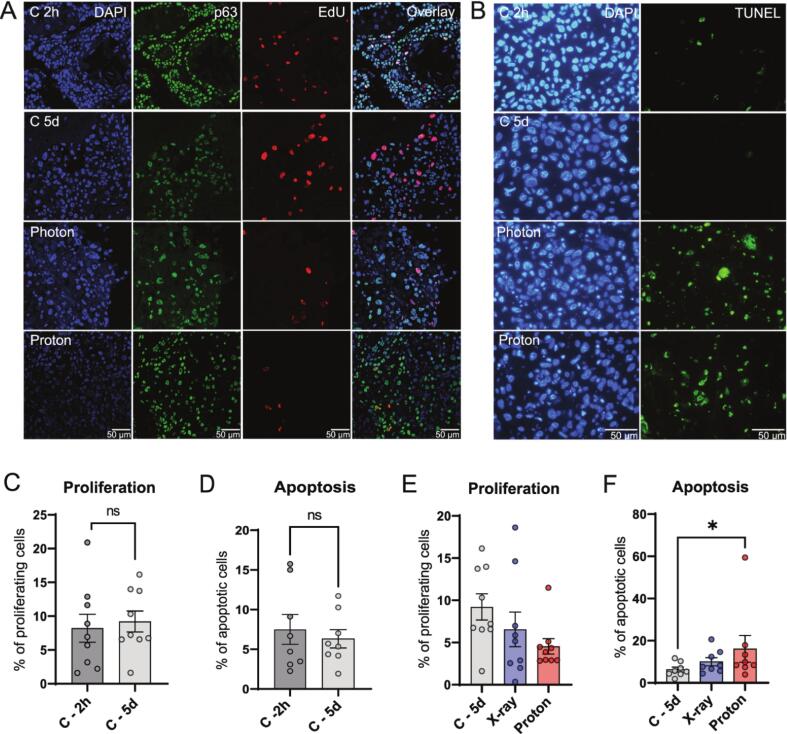
*Ex vivo* proliferation and apoptosis assays. (A) Representative confocal microscopy images of Edu incorporation of untreated control, 5 Gy X-ray and 5 Gy proton irradiated samples after 2 h and 5 days of culture (blue = DAPI for nuclei, green = p63, red = EdU; not RBE corrected). (B) Representative images of TUNEL staining of untreated control, 5 Gy X-ray and 5 Gy proton irradiated samples after 2 h and 5 days of culture (blue = DAPI for nuclei, green = TUNEL). (C) Percentage of proliferating cells in untreated control at the beginning of culture (2 h) and after 5 days of culture. (D) Percentage of apoptotic cells cells in untreated control at the beginning of culture (2 h) and after 5 days of culture. (E) Percentage of proliferating cells per treatment condition. (F) Percentage of apoptotic cells per treatment condition. Bar graphs represent mean of all samples (≥3 FoV per sample), and error bars depict SEM. Wilcoxon test (C, D) and Friedman test (E, F) were used for significance (paired values). *p < 0.05. (For interpretation of the references to colour in this figure legend, the reader is referred to the web version of this article.)

HNSCC tissue slices were irradiated either with X-ray, or in the horizontal proton beam as previously described (Supplementary Fig. 1A; [11]). A single dose of 5 Gy was used, as our previous study showed that this dose resulted in the largest heterogeneity in treatment response in HNSCC tumor samples [9]. Morphology of the untreated HNSCC tissue slices was preserved during culture (Supplementary Fig. 1B). Moreover, apoptosis (TUNEL assay) and proliferation (EdU incorporation) were constant for up to five days in culture for the majority of the samples (Supplementary Fig. 1C-F). Two samples increased in apoptosis by more than 10% in the absence of treatment and were therefore excluded from analysis (Supplementary Fig. 1G-H). Treatment outcome in the remaining samples showed on average decreased proliferation and increased apoptosis after both X-ray and proton irradiation after 5 days in culture, although only apoptosis induction after proton irradiation reached statistical significance (Fig. 1A–F).

### Identification of HNSCC samples specifically sensitive to proton irradiation

Next, we investigated whether there was a differential response to proton and X-ray irradiation in the individual HNSCC tumors. We compared the effect of X-ray and proton irradiation on tissue slices from the same tumor. For 5 samples, more than one individual slice of the same tumor was used per condition. Given no statistically significant differences in the percentage of EdU and TUNEL-positive cells between the individual slices in the majority of the samples (Supplementary Fig. 2), the FoV from the individual slices were pooled for further analysis.

**Fig. 2:**
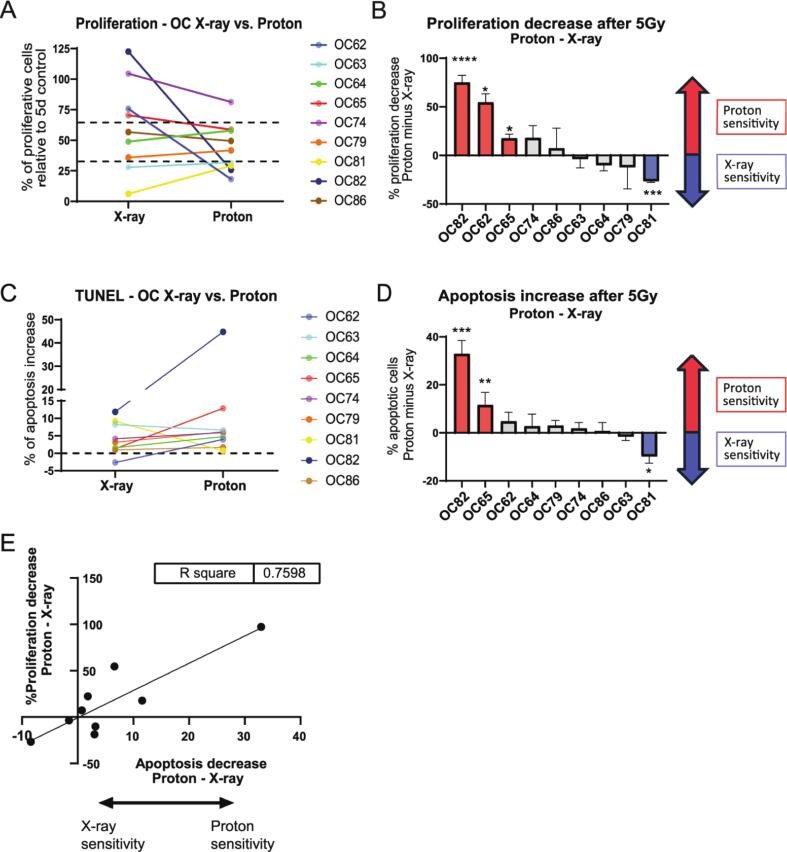
Comparison of X-ray and proton sensitivity. (A) Proliferation per sample relative to untreated control after X-ray or proton irradiation. (B) Difference in proliferation decrease upon proton or X-ray irradiation; decrease in proliferation was calculated relative to the experiment-specific control, followed by subtraction of the X-ray value from the proton value. Significance was calculated by directly comparing X-ray and proton irradiated samples. Sample numbers are displayed from high to low proton sensitivity. (C) Apoptosis induction (in percentage of apoptotic cells) per treatment condition, relative to untreated control. (D) Difference in apoptosis induction between proton and X-ray irradiation; experiment-specific control was subtracted from the irradiated condition, followed by subtraction of X-ray value from proton value. Significance was calculated by directly comparing X-ray and proton irradiated samples. Sample numbers are ordered from high to low proton sensitivity. (E) Correlation between the relative decrease of proliferation after proton irradiation (y-axis) and the relative induction of apoptosis after proton irradiation (x-axis). Bar graphs represent mean of all samples (≥3 FoV per sample; A,B,C,D), and error bars depict SEM (B,D). Kruskal-Wallis test with Dunn’s comparison was used for significance. *p < 0.05, ** p < 0.01, *** p < 0.001, **** p < 0.0001.

While the effects of both treatment modalities were comparable for most samples (n = 5), three tumors displayed a significantly larger decrease in proliferation upon proton than X-ray irradiation (Fig. 2A and B; Supplementary Fig. 3A). Moreover, two of these samples displayed markedly higher apoptosis levels upon proton irradiation (OC82, OC65; Fig. 2C and D; Supplementary Fig. 3B) Interestingly, one sample showed more severely decreased proliferation and increased apoptosis levels upon X-ray irradiation (OC81; Fig. 2A–D; Supplementary Fig. 3A and B). Overall, we found a positive correlation between the two measures of an increased proton sensitivity: the samples with the highest relative decrease of proliferation after proton irradiation displayed also the highest relative induction of apoptosis (R^2^ = 0.76; p = 0.0022, Fig. 2E).

### The HR-deficient sample displayed an increased proton radiation sensitivity *ex vivo*

**Fig. 3:**
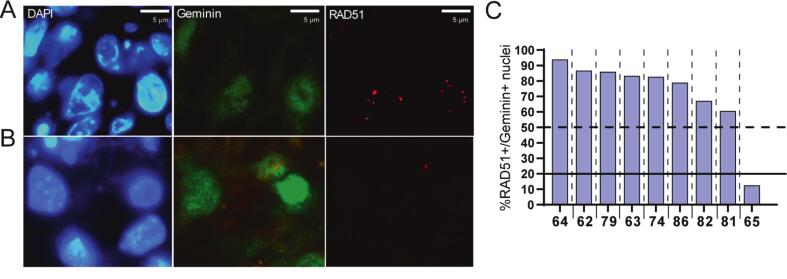
HR proficiency analysis. (A, B) Representative fluorescent images of RAD51 DNA damage foci of 5 Gy X-ray irradiated samples after 2 h for HR proficient (A) and HR deficient (B) sample (blue = DAPI for nuclei, green = geminin for cell cycle, red = RAD51). (C) RAD51 foci quantification two hours after irradiation. Geminin-positive cells were counted RAD51-positive when ≥ 5 RAD51 foci were present per nucleus. Samples were classified as proficient (above 50%), intermediate (20–50%) and deficient (under 20%). All bar graphs represent % of geminin positive cells (n ≥ 30) with 5 or more RAD51 foci. (For interpretation of the references to colour in this figure legend, the reader is referred to the web version of this article.)

We did not find a clear difference in the patient characteristics between proton- or X-ray sensitive HNSCC tumors (Supplementary Table 2). Therefore, the observed heterogeneity in the *ex vivo* treatment response points to tumor-intrinsic determinants, such as differences in the activity of DNA damage repair pathways. Studies suggest that the repair of proton-induced DNA damage is relatively more dependent on the HR DNA damage repair pathway [15], [16]. Therefore, we employed the REpair CAPacity (RECAP) assay to measure HR proficiency by quantifying RAD51 foci formation ([17] and Fig. 3A). Notably, only the proton-sensitive HNSCC sample OC65 was HR-deficient, while all the remaining samples were HR-proficient (Fig. 3B and C).

### HNSCC immune cell infiltration correlates with radiosensitivity *ex vivo*

**Fig. 4:**
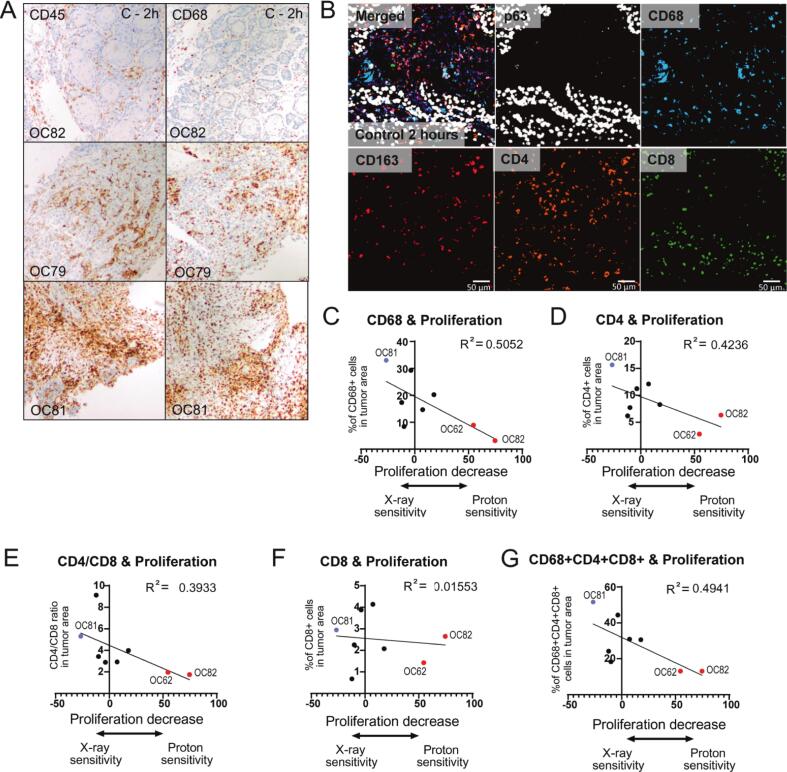
Immune cell infiltration and radiosensitivity *ex vivo*. (A) Representative brightfield microscopy image of immunohistochemistry staining (Blue (Hematoxylin = Nuclei, brown (DAB) = CD45 or CD68). (B) Representative fluorescent microscopy image of multiplex staining. (C-D) Correlation plots comparing immune cell infiltration to proliferation values after X-ray vs. proton irradiation for CD68 (general macrophages; C) and CD4 (T-helper cells; D). (E) Correlation plot for CD4/CD8 values. (F) Correlation plots for CD8 (cytotoxic T-cells). (G) Correlation plots comparing combined immune cell infiltration (CD68, CD4, CD8). Goodness of fit is displayed as R squared. Proton sensitive samples are marked in red (OC62, OC82), X-ray sensitive sample is marked in blue (OC81). (For interpretation of the references to colour in this figure legend, the reader is referred to the web version of this article.)

Further, we investigated whether differences in the immune cell infiltration could explain the heterogenous *ex vivo* proton and X-ray sensitivity. CD45 was used as a general marker for leukocytes and CD68 as macrophage marker. The X-ray radiosensitive sample OC81 displayed relatively high immune cell levels, while two of proton radiosensitive samples showed low levels of immune cell infiltration (OC82; OC62; Fig. 4A; Supplementary Fig. 4A).

To further define which immune cells could possibly explain the observed radiosensitivity, we performed multiplex staining detecting T-helper cells (CD4), cytotoxic T-cells (CD8), and macrophages (CD68), specifically M2 (tumor associated) macrophages (CD163; Fig. 4B). P63-positive areas were selected to directly define immune infiltration into tumor areas (Supplementary Fig. 4B; [18]). General macrophages (CD68+), T-helper cells (CD4+), the CD4+/CD8+ratio, as well as combined immune cells (CD68+, CD4+, CD8+) in tumor areas correlated with the treatment outcome *ex vivo*, with higher immune cell infiltration detected in the samples that were relatively more sensitive to X-ray irradiation (Fig. 4C–E and G; Supplementary Fig. 4C–E and I). This correlation was not observed for M2 macrophages or cytotoxic T-cells (Fig. 4 F; Supplementary Fig. 4F–H).

**Fig. 5:**
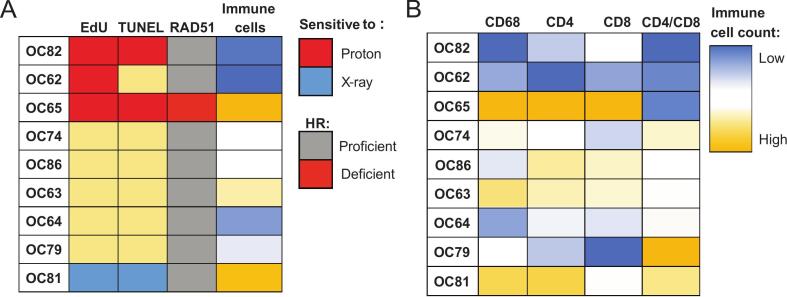
HNSCC microenvironment composition and HR deficiency correlate with radiosensitivity *ex vivo* (A) Schematic representation of tumor sensitivity, HR deficiency (RAD51 foci formation), and immune cell infiltration. For tumor cell sensitivity (based on proliferation (EdU, first column) and apoptosis (TUNEL, second column)), the relatively proton sensitive samples are depicted in red, the relatively X-ray sensitive sample in blue and the rest in yellow. The RAD51 foci deficient (HRD) sample is depicted in red and the relative level of immune cell infiltration as a gradient from low (blue) to high (yellow). (B) Immune cell infiltration per cell type (blue = low, yellow = high). Samples have been ordered from relatively high to low proton sensitivity based on proliferation (EdU incorporation) results. (For interpretation of the references to colour in this figure legend, the reader is referred to the web version of this article.)

The X-ray radiosensitive sample OC81 showed high immune cell infiltration and the proton radiosensitive samples OC62 and OC82 lacked immune cells in the tumor area. However, the proton radiosensitive sample OC65, that was HR-deficient, did not show the same trend. Moreover, not all immune cell low tumors showed an increased proton radiosensitivity (e.g. OC64; Fig. 5A). All three proton radiosensitive samples displayed a low CD4+/CD8+ T-cell ratio (Fig. 5B).

To conclude, we found in this limited sample set that immune cell infiltration correlated stronger to the general increased radiation response than to the specific proton radiosensitivity (Supplementary Fig. 3J–S).

## Discussion

HNSCC patient response to radiotherapy treatment is heterogeneous and cannot be explained by clinical factors only. In this study, we employed an *ex vivo* radiosensitivity assay to compare response of individual HNSCC tumors to proton and X-ray irradiation. We assessed the radiation response by combining proliferation and apoptosis assays, which have been described in literature as possible predictive assays for radiosensitivity [19], [20]. Three samples exhibited specifically increased sensitivity to protons, as compared to X-rays. One of the relatively more proton-sensitive samples was HR deficient and the other two showed very low immune infiltration, suggesting that these two parameters could determine the differential sensitivity.

For the comparison of proton and X-ray effects, a single dose of 5 Gy irradiation was used in the experimental setup. We have previously seen that at higher radiation doses (7 Gy and 10 Gy) the vast majority of HNSCC samples showed a very strong decrease in proliferation and therefore we reasoned that at those radiation doses any added effect of proton treatment would be lost. At 5 Gy we previously found the largest heterogeneity in response to X-ray irradiation and anticipated that this dose would be most suited to detect an increased sensitivity to protons in otherwise less (X-ray) responsive samples [9]. Indeed, three tumors specifically more sensitive to proton irradiation were detected (Fig. 2A).

We found a good concordance between a relative proliferation decrease and apoptosis induction after proton irradiation as compared with X-ray irradiation. However, the level of apoptosis after treatment, except for one proton-sensitive sample, remained low, consistent with previous observations [9]. Due to the previously observed large heterogeneity in the therapy response between individual HNSCC tumors, all samples were included in the analysis, even if they could be interpreted as outliers. When the one proton-sensitive sample with high apoptosis levels was excluded, the analysis still showed statistically significant increase of apoptosis after proton irradiation (p = 0.0485), Importantly, all the analyzed samples consistently displayed low level of apoptosis in the untreated condition after 5 days of culture. The optimal assay, or combination of assays, for determining radiosensitivity will require a larger clinical validation study. For X-ray irradiation, this is currently addressed in a prospective clinical study [21].

One of the samples with increased proton sensitivity *ex vivo* was HR deficient, in agreement with the hypothesis that the HR pathway is specifically required for repair of DNA damage induced by proton irradiation [15], [16], [22]. This has previously been suggested based on the increased proton sensitivity of HR deficient cell lines [23], [24], and our results in primary HNSCC tissue support this conclusion. The proton hypersensitivity of HR deficient cells and tumors may be the consequence of more clustered damage after proton irradiation, which may pose specific problems to DSB repair. This effect may be more pronounced at the distal end of the Bragg peak (where LET is higher) than in the SOBP, which would create heterogeneous responses throughout HR deficient tumors. More careful investigation will be necessary to address this issue directly in the proton-irradiated patients. Functional HR deficiency screening could identify patients that would specifically benefit from proton treatment. However, in our current and previously published cohort of HNSCC tumors the HR deficiency prevalence was low (<5%) [9], making this approach less relevant for the clinical practice.

Two of the X-ray resistant samples had very low immune cell infiltration, and the sample that was the most radiosensitive in general had high immune cell infiltration. According to the classification by Su et al. [25] this sample would most likely be categorized as immunoactive (type 1). This positive effect of an increased immune cell infiltration on X-rays sensitivity of tumors has been described previously [12], [26]. Conversely, several studies found that tumors with low immune cell infiltration showed increased radioresistance [25], [27]. Two out of three immunodeserted samples responded to protons, but not X-rays in our assay, suggesting that proton radiotherapy could be a better alternative for these patients. We are currently investigating whether a similar X-ray radioresistant effect is observed for the deserted immune type in an extended cohort of oropharyngeal squamous cell carcinoma (OPSCC) patients [21].

In our dataset, CD4+ T-helper cells as well as CD4+/CD8+ ratio seemed to be the most indicative of low *ex vivo* X-ray sensitivity. Low CD4+ T-helper cell infiltration has also been found to correlate with poor overall survival in HNSCC patients [28]. A low CD4+/CD8+ ratio has been shown to correlate in both cervical cancer and triple negative breast cancer with a worse 5-year survival rate [26], [29]. In the cervical cancer, both low CD4+/CD8+ ratio and low CD4+ T-helper cell infiltration correlated with an increase chance of patient’s death [29]. Although these immune parameters can be used to describe the response of a population, they do not appear to be sufficient to predict radiation responses of individual patients. Therefore, a functional assay may be crucial to define radioresistant patients on an individual level.

Most other *ex vivo* assays are costly and require extended culture time, making them less suitable for the clinical workflow [23], [24], [30]. Our MOTEX assay provides a rapid read-out of radiation response in less than 10 days, compatible with the clinical diagnostic timeframe. Moreover, the use of the complete tumor tissue preserves all cell types of the patient-specific tumor microenvironment, which appears to be specifically important for the sensitivity measurements that are dependent on immune components, such as X-ray sensitivity. However, there are also limitations to this assay. First, a minority of samples did not survive the experimental procedures optimally, which may be related to periods on ice and transport for irradiation at different locations (Erasmus MC and Holland PTC). Second, small differences between the experimental conditions at both locations (e.g. different incubators) cannot be excluded. Both issues could be solved by culturing and irradiating the tissue specimen with both X-rays and protons exclusively at the proton center. Through this change, diagnostic biopsy studies with smaller tissue specimens would become feasible.

In summary, our radiosensitivity assay was able to identify HNSCC samples that were specifically sensitive to proton irradiation, although the findings need to be confirmed in a larger group of patients. We are currently optimizing this assay for smaller tissue samples, which paves the way for the clinical studies, that would use biopsy material from a primary HNSCC tumor to predict specific radiosensitivity to protons *ex vivo*. After validation in a clinical study, this assay could be of value not only for personalized radiotherapy, but also as a platform for testing novel combination therapies.

## Institutional review board statement

The study was conducted in accordance with the Declaration of Helsinki and approved by the Ethics Committee of Erasmus MC (protocol code MEC-2017-1049 (2017)).

## Informed consent statement

Patient consent was waived for surgical material due to laws in the Netherlands for opting out. All use of human material was approved by the Erasmus MC Medical Ethics Commission.

## CRediT authorship contribution statement

**Katrin S. Pachler:** Data curation, Investigation, Writing – original draft. **Iris Lauwers:** Formal analysis, Visualization. **Nicole S. Verkaik:** Data curation, Investigation. **Marta Rovituso:** Resources, Writing – review & editing. **Hetty Mast:** Resources, Writing – review & editing. **Brend P. Jonker:** Resources, Writing – review & editing. **Bernd Kremer:** Resources, Writing – review & editing. **Sjors A. Koppes:** Resources, Writing – review & editing. **Thierry P.P. van den Bosch:** Resources, Writing – review & editing. **Gerda M. Verduijn:** Writing – review & editing. **Steven Petit:** Writing – review & editing. **Brita S. Sørensen:** Writing – review & editing. **Dik C. van Gent:** Conceptualization, Methodology, Project administration. **Marta E. Capala:** Conceptualization, Methodology, Project administration.

## Declaration of competing interest

The authors declare the following financial interests/personal relationships which may be considered as potential competing interests: Dr. Marta Capala has received consulting fees for consultancy for GE Healthcare on behalf of the Department of Radiotherapy, Erasmus Medical Center Rotterdam, though not related to the research described in the manuscript.

Prof. Brita Singers Sørensen is an Editorial Board Member with Radiotherapy and Oncology and was not involved in the editorial review or the decision to publish this article.

Erasmus MC Cancer Institute has research collaborations with Accuray Inc., Sunnyvale, USA, Elekta AB, Stockholm, Sweden and with Varian, a Siemens Healthineers Company (Palo Alto, CA, USA).

## Data Availability

All data can be found in the manuscript.
