# Supplementary material for: Motion-based tissue ex vivo (MOTEX) assay to assess proton and X-ray irradiation responses in head and neck squamous cell carcinoma
Source: Clin Transl Radiat Oncol. 2026 Feb 8;58:101124. doi: 10.1016/j.ctro.2026.101124 (PMC12930084; doi:10.1016/j.ctro.2026.101124)
Supplement: Supplementary Data 1 [file mmc1.docx]

Supplementary Materials & Methods

2.9 Multiplex Immunofluorescence

In brief, following deparaffinization and heat-induced antigen retrieval with CC1 (#950-224, Ventana) for 32 min, anti-CD4 was incubated for 32 min at 37 °C followed by omnimap anti-rabbit HRP (#760-4311, Ventana) and detection with R6G (#950-240, Ventana). An antibody denaturation step was performed with CC2 (#950-123, Ventana) at 100 °C for 20 min. Secondly, incubation with either anti-CD68 was performed for 32 min at 37 °C, followed by Omnimap anti-mouse (#760-3210 Ventana) and detection with detection with DCC (#760-244, Ventana). An antibody denaturation step was performed with CC2 (#950-123, Ventana) at 100 °C for 20 min. Secondly, incubation with either anti-CD163 was performed for 32 min at 37 °C, followed by Omnimap anti-mouse (#760-3210 Ventana) and detection with Red610 (#760-245, Ventana) for 8 min. An antibody denaturation step followed with CC2 at 100 °C for 20 min. An antibody denaturation step followed with CC2 at 100 °C for 20 min. Fourthly, anti-p63 was incubated for 4 minutes at 37 °C, followed by omnimap anti-rabbit HRP (#760-4311, Ventana) and detection with detection with Cy5 (#760-238, Ventana) for 8 min. An antibody denaturation step followed with CC2 at 100 °C for 20 min. Lastly, anti-CD8 was incubated for 4 minutes at 37 °C, followed by omnimap anti-rabbit HRP (#760-4311, Ventana) and detection with detection with FAM (#760-243, Ventana) for 8 min. Finally, slides were washed in phosphate-buffered saline and mounted with Vectashield containing 4’,6-diamidino-2-phenylindole (Vector laboratories, Peterborough, UK). Slides were imaged with Axioscan Zeiss.

| Antibody | Dilution | Species | Company | Clone | Ab incubation  time at 37˚C |
| --- | --- | --- | --- | --- | --- |
| CD4 | 2,05 ug/ml | rabbit | Ventana | SP35 | 32 minutes |
| CD8 | 1,04 ug/ml | mouse | DAKO | C8/144B | 32 minutes |
| CD68 | 3,49 ug/ml | mouse | Ventana | KP1 | 20 minutes |
| CD163 | 1,09 ug/ml | mouse | Cell Marque | MRQ-26 | 20 minutes |
| P63 | 1,04 ug/ml | rabbit | Cell Marque | EP174 | 32 minutes |

**Supplementary Table 1:** Antibody details used for multiplex immunofluorescence staining.


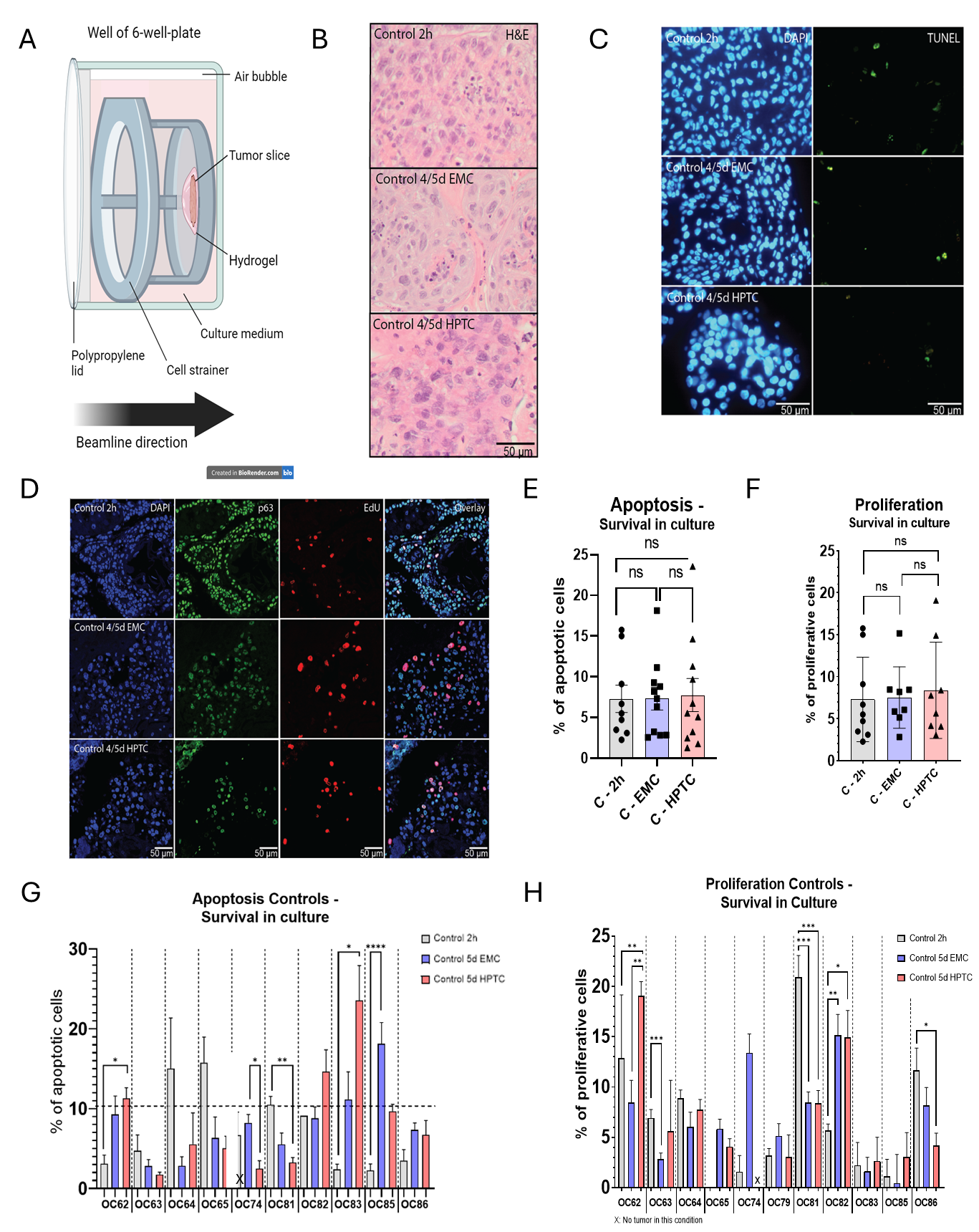


**Supplementary Figure 1: *Ex vivo* tissue slice cultures and proton irradiation.** (A) Schematic image of proton setup. Representative H&E staining (B), fluorescent TUNEL (C) and EdU staining (D) at day 0 and day 4/5 of ex vivo culture at both EMC and HPTC locations. (E) Mean of apoptotic cells (percentage) of control conditions at the beginning (2 hours) and after 4-5 days of culture. (F) Mean of proliferating cells (percentage) of control conditions at the beginning (2 hours) and after 4-5 days of culture. (G) Percentage of apoptosis in control conditions at day 0 and day 4-5 of culture. (H) Percentage of proliferation in control conditions at day 0 and day 4-5 of culture. Samples are ordered by sample number. Bar graphs represent mean of all samples (≥3 FoV per sample), and SEM is depicted as error bars. Kruskal-Wallis and Dunn’s multiple comparison test was used for significance. * p < 0.05, ** p < 0.01, *** p < 0.001, **** p < 0.0001.


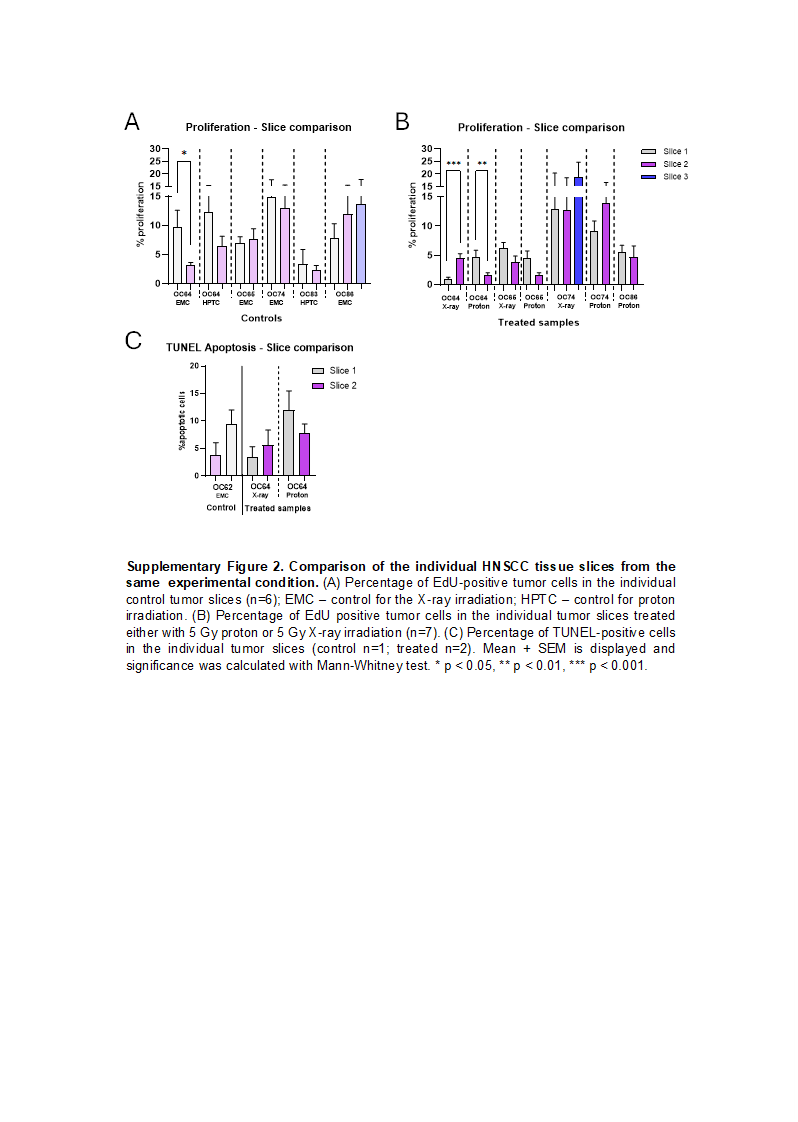


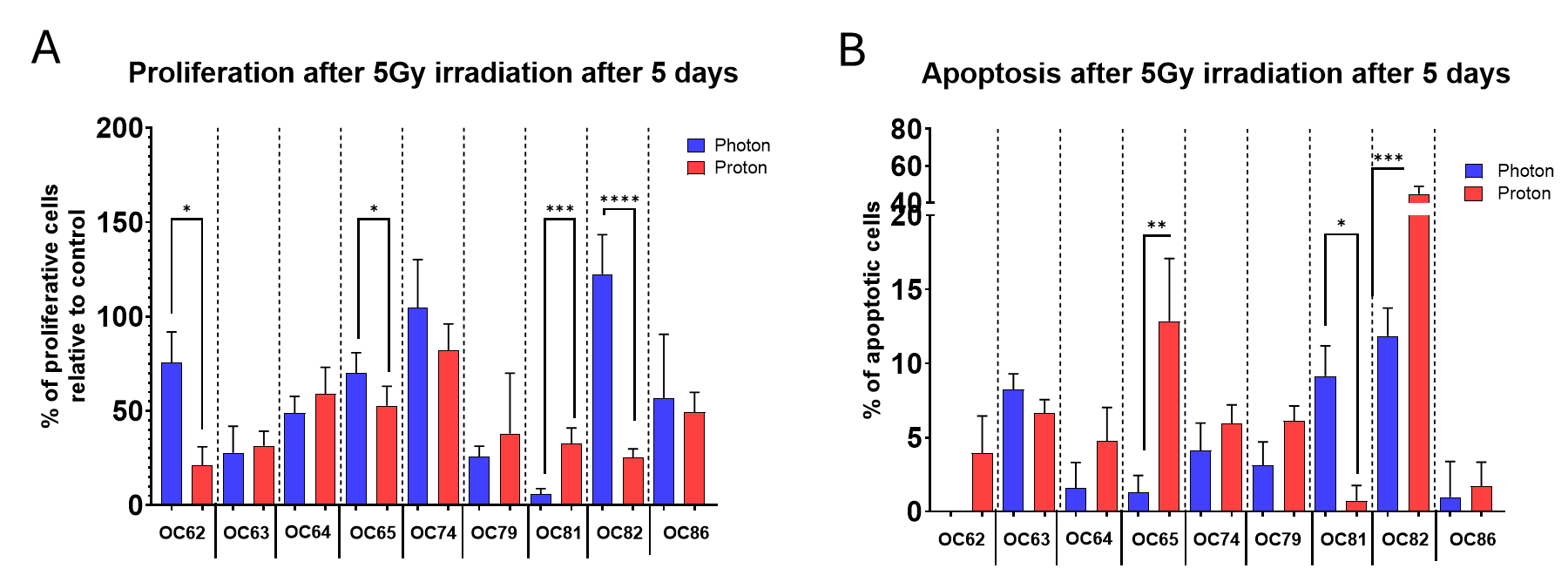


**Supplementary Figure 3: Identification of HNSCC samples specifically sensitive to proton irradiation.** Percentage of apoptotic cells (A) and proliferating cells (B) in 5Gy X-ray and 5Gy proton irradiated samples after 4-5 days of culture. Graph A displays proliferation values after irradiation relative to controls. Graph B displays increase in apoptosis after irradiation compared to controls. Bar graphs represent mean of all samples (≥3 FoV per sample), and SEM is depicted as error bars. Kruskal-Wallis and Dunn’s multiple comparison test was used for significance. * p < 0.05, ** p < 0.01, *** p < 0.001, **** p < 0.0001.


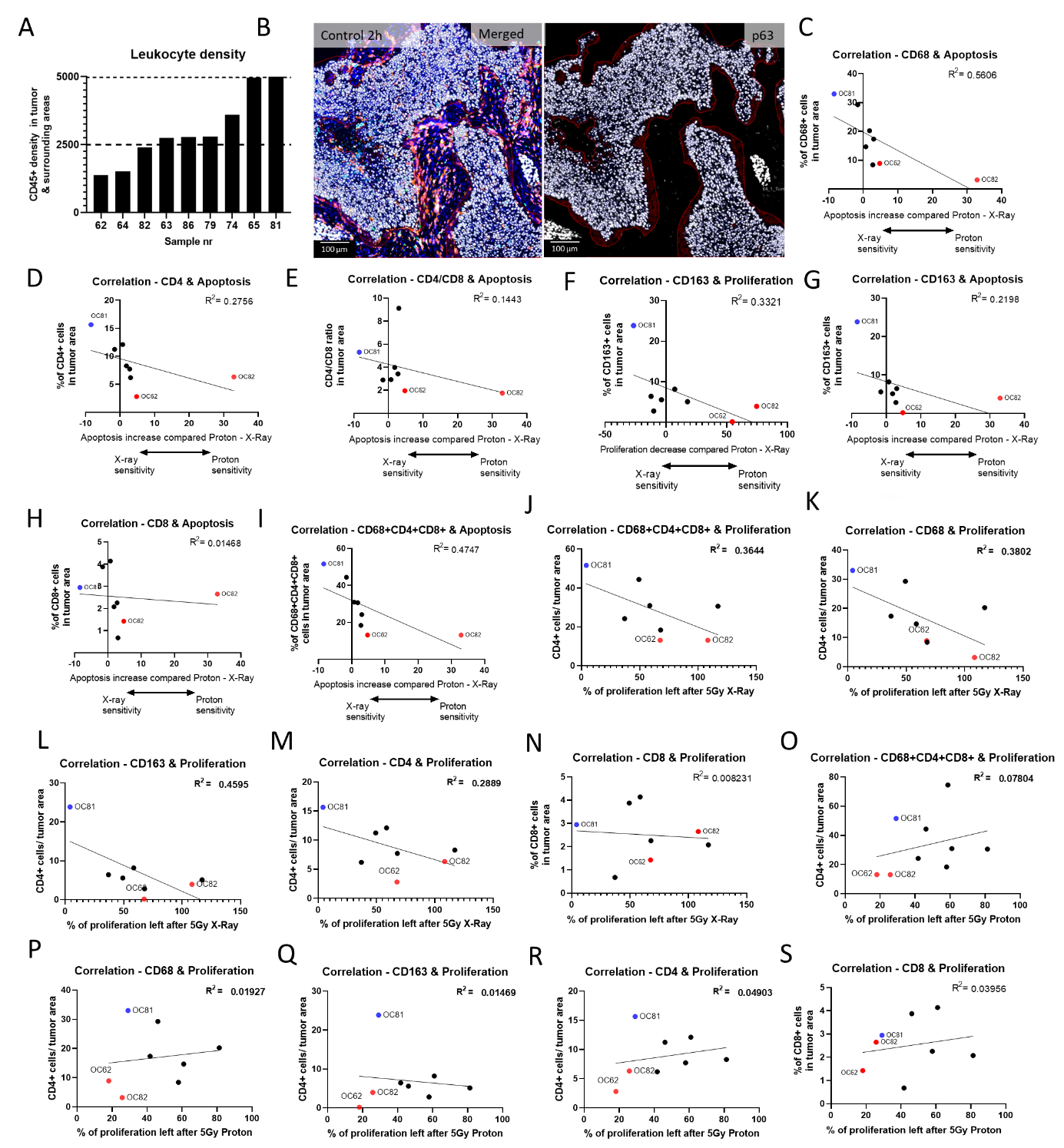


**Supplementary Figure 4: HNSCC immune cell infiltration correlates with radiosensitivity *ex vivo*.** (A) Leukocyte (CD45+) density in tumor and close surrounding areas per mm^2^ calculated from whole slide IHC scans. (B) Representative fluorescent multiplex image to showcase p63+ area selection (White = p63+ nuclei). (C-E) Correlation plots comparing immune cell infiltration to apoptosis values after X-ray vs. proton irradiation for CD68 (general macrophages; C) CD4 (T-helper cells; D) and CD4/CD8 ratio (E). (F-G) Correlation plots comparing immune cell infiltration to proliferation and apoptosis values after X-ray vs. proton irradiation for CD163 (M2 macrophages). (H) Correlation plot for apoptosis values X-ray vs. proton for CD8 (cytotoxic T-cells) and CD68+CD4+CD8+. (J-N) Correlation plots for percentage of immune cell subtypes (CD68+CD4+CD8+, CD163, CD68, CD4, CD8) and remaining proliferation after 5Gy X-ray. (O-S) Correlation plots for percentage of immune cell subtypes (CD68+CD4+CD8+, CD163, CD68, CD4, CD8) and remaining proliferation after 5Gy proton. Proton sensitive samples are marked in red (OC62, OC82), X-ray sensitive sample is marked in blue (OC81).

| **Sample number** | **Age** | **Gender** | **Tumor stage** | **Tumor size (mm)** | **Smoking (YES/NO)** | **Alcohol (YES/NO)** | **Distant metastasis (YES/NO)** |
| --- | --- | --- | --- | --- | --- | --- | --- |
| OC62 | 77 | F | pT3N1 | 35 | N | N | N |
| OC63 | 56 | F | pT2N0 | 28 | N | N | N |
| OC64 | 75 | M | pT4aN0 | 32 | N | Y | N |
| OC65 | 61 | F | pT4aN2b | 21 | N | N | N |
| OC74 | 80 | F | pT1N0 | 6 | N | Y | N |
| OC79 | 46 | M | pT3N2b | 14 | N | Y | N |
| OC81 | 62 | F | pT3N0 | 35 | N | N | N |
| OC82 | 77 | M | pT4aN0 | 30 | Y | N | N |
| OC86 | 51 | M | pT4aN1 | 25 | Y | Y | N |

**Supplementary Table 2: HNSCC microenvironment composition and HR deficiency correlate with radiosensitivity *ex vivo*** (A) Table displaying patient characteristics ordered by OC number. Displayed is age, gender, tumor stage, tumor size in mm, current smoking status, frequent alcohol intake and distant metastasis.
